# Transitional forms between the three domains of life and evolutionary implications

**DOI:** 10.1098/rspb.2011.1581

**Published:** 2011-09-14

**Authors:** Emmanuel G. Reynaud, Damien P. Devos

**Affiliations:** 1 School of Biology and Environmental Science, UCD Science Centre, Belfield, Dublin 4, Ireland; 2 European Molecular Biology Laboratory, Meyerhofstrasse 1, 69117 Heidelberg, Germany

**Keywords:** eukaryotic origin, archaea, evolution, transition forms, platypus

## Abstract

The question as to the origin and relationship between the three domains of life is lodged in a phylogenetic impasse. The dominant paradigm is to see the three domains as separated. However, the recently characterized bacterial species have suggested continuity between the three domains. Here, we review the evidence in support of this hypothesis and evaluate the implications for and against the models of the origin of the three domains of life. The existence of intermediate steps between the three domains discards the need for fusion to explain eukaryogenesis and suggests that the last universal common ancestor was complex. We propose a scenario in which the ancestor of the current bacterial Planctomycetes, Verrucomicrobiae and Chlamydiae superphylum was related to the last archaeal and eukaryotic common ancestor, thus providing a way out of the phylogenetic impasse.

## 1. Introduction

*Transitional forms*. Charles Darwin was well aware that his theory of descent with modification required the existence of transitional forms between species; indeed, the lack of such forms was one of the main arguments initially used by its opponents. Fortunately, the discovery of the first such fossil was reported shortly after the publication of ‘On the origin of species’, thus dealing a fatal blow to this criticism. Darwin did not directly address the division of the tree of life (ToL) into three branches—archaea, bacteria and eukaryotes. However, the paucity of intermediary forms is similarly used to argue against continuous evolution in the deepest branching points of the tree [1].

The three domains share a set of features, including an RNA polymerase, ribosomes, membrane protein insertion systems and a common genetic code, asserting their common ancestry to the last universal common ancestor (LUCA). Each domain also has unique features developed after divergence from the common ancestor [2], justifying their classification in different groups. In addition, archaea and bacteria share a set of fundamental characteristics that includes the 5S, 16S and 23S rRNAs, a circular chromosomal architecture with genes arranged in operons, and capless translational initiation. Archaea also have an evolutionary link with eukaryotes through their gene-expression machinery and there is increasing support that eukaryotes and archaea shared a common ancestor at the exclusion of bacteria, the last archaeal and eukaryotic common ancestor (LAECA) [3,4]. However, a bigger proportion of eukaryotic genes are related to bacterial ones than to archaeal ones [5,6].

*Phylogenetic impasse*. Reconstructing the early steps of life is still controversial. Whether the LUCA is a simple cellular entity, a sophisticated and genetically diversified organism or even a proto-eukaryote from which the three domains have then emerged mainly by reductive evolution is the subject of much debate [7–9]. Various scenarios on the origin of the three domains have been proposed [1,9]. The controversy is best exemplified by a recent comparison of various analyses focusing on the relationship between archaea and eukaryotes. This study concluded that minor variations in the dataset, the taxonomic sampling or the analytical method could cause marked differences in the results [3]. Thus, such tools may be poorly suited to infer relationships so deep in the ToL as supported by theoretical studies [10]. An important concern was raised regarding the interpretation of results, which seemed to be based mainly on subjective interpretation to support a preconceived scenario favoured by the authors. It was concluded that we are currently located in a phylogenetic impasse [3].

*Intermediate steps out of the phylogenetic impasse*. Recently, the identification of features previously thought to be specific to eukaryotes or archaea has been reported in some members of the bacterial Planctomycetes, Verrucomicrobiae and Chlamydiae (PVC) superphylum. Here, we review these features, and evaluate their implications for our understanding of the origins of the three domains of life. We argue that the PVC features represent intermediate steps between the three domains and that the presence of such features in a bacterial superphylum suggests a scenario in which the ancestor of the current PVC members was related to the LAECA. This in turns supports that the eukaryotic endomembrane system evolved by internalization of the bacterial periplasm.

*The PVC superphylum*. Genetic, biochemical and rRNA data clearly locate the PVC members in the bacterial domain. The PVCs are consistently recovered as a monophyletic group in phylogenetic trees based on a variety of methods and datasets, but those analyses are inconclusive as to the relationship of PVC members with the other bacterial groups [11–25]. Challenges posed by the compartmentalization of planctomycetes have been outlined [26].

## 2. Eukaryotic and archaeal features in Planctomycetes, Verrucomicrobiae and Chlamydiae members

The similarities between PVC members that lend support to the monophyly of the group include features that are uncommon for bacteria and usually considered as eukaryote- or archaea-specific. These features are summarized below (note that not all features are found in all PVC members).

*Complex cell plan*. The major difference between most Gram-negative (G(−)) bacteria and PVC members is that the cytoplasmic membrane is invaginated, sometimes extensively, in the cytoplasm to define different types of cellular organization [27,28]. Our previous publication [29] and ongoing work demonstrate that the PVC outer and innermost membranes are not different from the outer and inner membranes of G(−) bacteria, and that the space between them (called paryphoplasm) is equivalent to the periplasm. This is ultimately demonstrated by the fact that, like in other bacteria, ribosomes line up against the inner membrane and, in PVC members, its invaginations. The main difference is that the PVC periplasm is usually larger with a more complex organization than the ‘classical’ bacterial periplasm. This feature is shared between PVC members but shows important variations [28,30]. In the planctomycete *Gemmata obscuriglobus*, the invaginations and derived membrane morphologies appear to be dynamic and cell cycle-dependent [29]. The presence of this feature in most PVC members suggests that the ancestor of the PVC supergroup already had this feature [28]. In addition, it has been claimed that the *G. obscuriglobus* surrounds its genomic DNA with a folded single membrane, topologically similar to the eukaryotic nuclear envelope [31]. It is, however, unclear whether this membrane completely surrounds the DNA and detailed three-dimensional studies of this planctomycete are needed to solve this important issue.

*Membrane coats*. Membrane coats (MCs) are proteins that play key roles in shaping eukaryotic membranes. Most MCs exhibit a unique arrangement of beta-propeller and alpha-helical repeat domains [32] thought to be exclusive to eukaryotes until their discovery in various PVC members [29]. In addition, MCs have been associated with membrane manipulation and endocytosis (see below) in the planctomycete *G. obscuriglobus*. No signs of a recent horizontal gene transfer (HGT) to or from the bacteria could be detected leading to the suggestion that the bacterial PVC superphylum contributed to the origin of the eukaryotic endomembrane [29]. A more complete characterization of the proteins involved in the definition of the PVC endomembrane system would bring important answers.

*Condensed DNA*. Like the eukaryotic genomic material, the nucleoids in PVC members appear condensed when cryofixed and cryosubstituted [27,28] which, unlike conventional chemical fixation, is not expected to yield such condensation as an artefact. This contrasts with the appearance of cryofixed nucleoids from other bacterial species such as *Escherichia coli* and *Bacillus subtilis*, where an irregularly shaped nucleoid extends through the cell cytoplasm. Several proteins related to eukaryotic chromatin-associated ones are present in the *Chlamydia trachomatis* genome, suggesting a eukaryotic-like mechanism for chlamydial nucleoid condensation and decondensation [33]. *Chlamydiae* are also one of the few prokaryotic organisms reported to contain proteins homologous to eukaryotic histone H1, although the similarity might be biased by the low complexity of the protein [34–36]. However, owing to their parasitic lifestyle, the possibility of HGT is difficult to rule out. Isolation of the factors involved in condensing the PVC DNA will be an important step forward [37].

*Budding division*. Planctomycetes are one of the few groups of bacteria that reproduce by budding [38]—a mode of division more commonly associated with eukaryotes.

*Sterol*. Sterols and related compounds play essential roles in the physiology of eukaryotic organisms, including the regulation of membrane fluidity and permeability. Sterols are almost completely absent in prokaryotes but are nearly ubiquitous in eukaryotes. The origin of sterol biosynthesis is still debated but it is accepted that the early eukaryote could synthesize a large array of different sterols [39]. Among the few bacteria that synthesize sterol, *G. obscuriglobus* contains the most abbreviated sterol pathway identified in any organism; its major products are lanosterol, a simple sterol and its uncommon isomer, parkeol [40]. The primitive sterols suggest that this genus has retained an ancient sterol biosynthetic pathway. No evidence of HGT was found in planctomycetes. A definitive phylogenetic analysis of the PVC sterol synthesis pathway would bring invaluable clues to this important process.

*Lipids*. The membranes of planctomycetes contain lipids that are more typical of eukaryotes, such as palmitic, oleic and palmitoleic lipids [41]. One of the distinguishing features of archaea is the presence of ether-linked lipids rather than the ester-linked lipids found in bacteria. Eukaryotes contain both types of lipid. How these lipid pathways evolved is a fundamental question in biology [42,43]. Anammox planctomycetes have a variety of unusual lipids, and are the only prokaryotes having both ether- and ester-linked lipids in their membranes, and could thus be considered a transition point for this feature between archaea and bacteria [44]. Determining the cellular localization of the different lipid classes and how they coexist would have tremendous evolutionary implications.

*Methane cycle*. C1 transfer chemistry is at the core of two important reactions central to the Earth's methane balance, methanotrophy and methanogenesis. The origins of both are still unknown. Methanotrophs are mainly found in the alpha-, beta- and gamma-proteobacteria as well as in some archaea and methanogens in the archaeal domain Euryarchaeota. Planctomycetes have also been shown to contain C1 transfer genes [45,46]. Ancient divergence of Verrucomicrobia and Proteobacteria C1 genes has been recognized [47] but their phylogenetic position is still disputed [45,46,48]. This prompted the statement that ‘Planctomycetes may hold a key to the origins of methanogenesis and methylotrophy’ [46]. Determining the enzymatic activity of the PVC C1 enzymes would be revealing in this respect.

*Cell wall*. Peptidoglycan is a standard component of almost all bacterial cell walls but is absent from the cell walls present in many eukaryotes and archaea. The peptidoglycan synthesis genes are contained in the division and cell wall (*dcw*) gene cluster that is highly conserved in bacteria. However, the *dcw* gene cluster shows alteration in most PVC members and is almost completely absent in some of them [16]. In addition, the cell wall of various Planctomycetes, like eukaryotes, is mainly composed of proteins [41].

*FtsZ and tubulin*. Bacterial cell division relies on concentric rings of the FtsZ protein. FtsZ is found in most bacteria and in one subdivision of the archaea, the Euryarchaeota, whereas its homologue, the cytoskeleton protein tubulin, is usually restricted to eukaryotes. Unlike most bacteria, some PVC members show alteration of the *ftsZ* gene, while it is absent in Chlamydiae and Planctomycetes [16,25]. Some Verrucomicrobia have both tubulin and FtsZ homologues encoded in their genome; a situation unique among all forms of life [49–51]. It is not clear if the presence of tubulin in this bacterium is the result of HGT or of a deep evolutionary connection. However, the Verrucomicrobia tubulins do not branch within the eukaryotic ones in phylogenetic trees, but instead behave as an outgroup, arguing against HGT from a modern eukaryote [49]. In addition, the *Prosthecobacter* genes represent an intermediate step between FtsZ and tubulin, in terms of both structure and folding [51–54]. One clue to understand those unique PVC features is that the essentiality of the *ftsZ* gene is most probably linked to the presence of a peptidoglycan cell wall [55–58].

*Endocytosis*. Key to eukaryotic evolution was the development of endocytosis, the process by which cells absorb molecules such as proteins from outside the cell by engulfing them with their cell membrane. Phylogenetic analysis suggests that the endocytic molecular machinery must have been present in the last eukaryotic common ancestor (LECA) [59]. Unexpectedly, a related process has now been described in the planctomycete *G. obscuriglobus* [60]. This process is linked to MC-like proteins, and is energy-dependent and receptor-mediated, rendering it similar to eukaryotic endocytosis. Determining the players involved in this process would thus be extremely important.

*The last PVC common ancestor*. The presence of the above characteristics in a diffuse pattern throughout the members of the PVC superphylum suggests that the LPCA had most of these features and some were subsequently lost during divergence of the phyla. Additional sampling of the PVC superphylum will undoubtedly refine our perception of the LPCA and its characteristics.

*Other bacteria with eukaryotic or archaeal features*. PVC superphylum members are not the only bacteria to display archaeal- or eukaryotic-like features. However, compared with other bacterial features and disregarding those that are due to HGT, the PVC trait is often the one that is most ‘similar’ to the corresponding eukaryotic or archaeal ones. For example, the endomembrane vesicles found in *Rhodobacter* are mostly protein dominated [61] and not sustained by MC-like proteins, like those found in PVC members. In addition, the PVC superphylum is the only one combining so many of these features in related species.

## 3. Discussion

*Evolutionary relationship*. Evaluating the evolutionary relationship of these particular bacterial features is a difficult task owing to the dominant lack of sequence similarity between PVC proteins and their non-bacterial counterparts. The paucity of sequence information raises the possibility that any similarities observed may be the result of misinterpretation or, at best, convergence. If so, a thorough characterization of the PVC features will still provide invaluable insight into the alternative development of those features.

Although HGT can be invoked on a case-by-case basis, a global view argues against such considerations. Firstly, this possibility has been investigated in several cases, i.e. the C_1_, MC and tubulin genes. In none of them could the occurrence of HGT be unambiguously demonstrated, most explanations instead favoured an ancient vertical relationship, albeit without unequivocally establishing it [29,40,45,46,49–51]. Secondly, while HGT is possible for traits involving few genes, such as sterol synthesis, this is improbable for more complex features. For example, membrane organization is unlikely to be achieved by the transfer of several MC-coding genes alone. The same can be said for genome compaction and probably for the majority of eukaryotic and archaeal features. Similarly, although convergence could explain some features, it is unlikely to explain all of them.

On the other hand, the lack of sequence similarity does not necessarily imply a lack of homology, as demonstrated by the bacterial and eukaryotic cytoskeleton proteins, MreB/Actin and FtsZ/Tubulin [62]. In fact, despite the lack of sequence similarity, some PVC traits seem to be intermediate between bacterial and non-bacterial features [63]. In addition, aspects of several features can be interpreted as signs of homology followed by divergence of the coding sequences. For example, tertiary structure and function similarities link the bacterial and eukaryotic MC proteins [29,60], as predicted by the protocoatomer hypothesis [32].

Despite remaining currently unproven, a possible vertical descent relationship between the PVC and archaeal or eukaryotic features is important to consider because it provides a way out of the phylogenetic impasse.

*Intermediate steps*. First and foremost, the presence of eukaryotic and archaeal features in bacteria demonstrates the existence of intermediate forms between the three domains of life [63]. Thus, this observation definitively disproves the argument that the lack of intermediates rejects the gradual evolution of eukaryotic traits. It follows that the presence of both eukaryotic and archaeal features in bacteria discards the requirement for fusion to explain eukaryogenesis. In addition, a fusion scenario involving PVC members has already been evaluated and rejected elsewhere because it still requires *ad hoc* assumptions and fails to convincingly explain the origin of most features [64]. The presence of such eukaryotic and archaeal features in a bacterial superphylum agrees with a previously suggested complex LUCA [9]. This LUCA might have displayed features such as sterol production, endocytosis and a complex membrane organization based on MC proteins, among others. However, reductive evolution from a complex LUCA requires a substantial amount of losses in the three domains. In addition, given the undoubted bacterial nature of PVC members, it is most likely that the LPCA was already established as a bacterium.

### (a) The cauldron hypothesis

We suggest an alternative scenario of a ‘classical’ bacterial rooting of the ToL in which the LPCA was a sister entity to the LAECA. Their common ancestor would have served as a ‘cauldron’ for the evolution of eukaryotic and archaeal features. Similar to the platypus that exhibits a combination of characteristics that are a legacy of the common ancestor shared between birds, reptiles and mammals, the archaeal and eukaryotic features found in PVC members might reflect a common ancestor between bacteria and the LAECA. In this LPCA-based scenario, the features found in the LPCA are ancestral to the eukaryotic and archaeal ones, and also to the current PVC ones. The features found in current PVC members are then derived from the LPCA ones and are not ancestral to the eukaryotic or archaeal features. Thus, an ancestry signal for the PVC proteins or genes when compared with the archaeal and eukaryotic ones is not a requirement of this scenario.

The LPCA sisterhood relationship to the LAECA also provides a credible transition point for the appearance of the archaeal membrane in anammox, the only prokaryote known to date to have both ester- and ether-linked lipids. It is possible that the LAECA had both ether- and ester-linked lipids, which were retained in eukaryotes while the ester linkage was lost in archaea. Determining the stereochemistry (another major difference between archaeal and eukaryotic and bacterial lipids) of the anammox lipids would contribute important information to this issue.

It can be argued that genomic comparison does not reveal a strong link between eukaryotes and planctomycetes [65]. The main counterargument is that this only illustrates the limits of sequence-only based methods. This is supported by the discrepancies observed for phylogenetic investigation of the eukaryotic–archaeal relationship, the monophyly of the PVC superphylum itself, and its relationship with the other bacterial groups. The detection of MCs in PVC members clearly demonstrates this point, as the similarity reported could only be detected through structural analysis and not from sequence-only searches [29].

### (b) Implications of the hypothesis

Perhaps the strongest argument in favour of this scenario is that it provides an intermediate step supporting the hypothesis of the origin of the eukaryotic endomembrane system by internalization of the bacterial periplasm [66].

*Internalization of the bacterial periplasm at the origin of the eukaryotic endomembrane system*. The development of the endomembrane system has been crucial to eukaryogenesis and there is growing evidence that the early eukaryote already possessed a complex endomembrane system [67–69]. The eukaryotic endomembrane system plays a role in the filtering and degrading of external compounds that enter the cell as well as in the externalization of cellular products. Similarly, the bacterial periplasm can be considered as a buffer between the outside and the inside of the cell. Functional similarities between the bacterial periplasm and the eukaryotic endomembrane system, including protein import mechanisms [70], chaperones [71], the unfolded protein response [72], the presence of outer membrane vesicles [73], multi-drug resistance efflux pumps [74] and kinases [75], suggest that they have related functions and that the latter is the result of the internalization of the former by invaginations of the inner membrane [66]. However, this proposal has so far failed to gather much support as no intermediate forms have been described.

**Figure 1.**
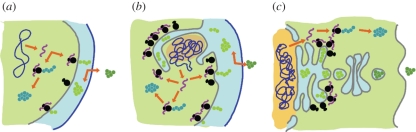
Intermediate step of bacterial periplasm internalization in the Planctomycetes, Verrucomicrobiae and Chlamydiae superphylum. Schematic of the cellular organization found in (*a*) bacteria, (*b*) *G. obscuriglobus* planctomycete and (*c*) eukaryotes. Proteins are represented by strings of beads; cytoplasmic ones are blue-green, excreted ones are dark green, peri- or endoplasmic ones are light green. DNA is purple and ribosomes are black. Cytoplasm is green, endoplasm is blue and nucleoplasm is orange. Outer membrane is dark blue, cytoplasmic or plasma membrane is grey.

In the LPCA scenario, PVC members provide these intermediate forms (figure 1). Anammox planctomycetes carry out the anaerobic oxidation of ammonium in an anammoxosome, which is fully separated from the periplasm, and therefore represents an endoplasmic compartment in which specific functions occur [76]. This is similar to eukaryotic organelles in which specific functions are physically separated from the cytoplasm by a membrane. The PVC inner membrane invaginations are lined with ribosomes, most probably targeting proteins to the lumen, in an organization reminiscent of the rough endoplasmic reticulum. Vesicle-like structures are also observed in the cells of *G. obscuriglobus* [29]. This particular cell plan is shared with Verrucomicrobia and Lentisphaera members and thus, was probably present in the LPCA [28]. Importantly, eukaryotic-like MCs are involved in this bacterial endomembrane system [29,60]. A similar exception to the prokaryotic cell plan has been described in the archaea *Ignicoccus hospitalis* [77], additionally supporting this scenario [78]. Interpreted in a bacterial rooting of the ToL, there is thus a membranous continuum from the bacterial periplasm via the PVC paryphoplasms to the eukaryotic endomembrane system (figure 1). Internalization of inner membrane invaginations is topologically coherent with a nuclear envelope that has the appearance of a double membrane but is in fact a folded single one. Hence, the various organizations of the PVC membranes support the proposal that the eukaryotic endomembrane system evolved by internalization of the bacterial periplasm by demonstrating the existence of intermediate forms.

Assuming a bacterial rooting of the ToL, one important step of eukaryogenesis is the loss of the outer membrane [79]. This has previously been criticized by arguing that the functionality of the periplasm would then be lost. This argument is invalidated here by the proposal that this step will only happen once the periplasm and its functionalities have been internalized.

*From the eukaryote–archaea ancestor to mitochondrial acquisition*. The internalization of the periplasm is also consistent with mitochondrial acquisition scenarios, in which mitochondria are derived from parasites and symbionts that were slowly adapted [80], as most parasites and symbionts are located in the periplasm and would therefore also be internalized. This is supported by the fact that the mitochondria present in the first eukaryotic cell has evolved considerably since the alpha-proteobacterium that first entered in contact with the eukaryotic ancestor [81]. The generic presence of mitochondria in eukaryotes demonstrates the simultaneous appearance of an endomembrane system and mitochondria during eukaryogenesis. A similar explanation applies to the origin of the chloroplast and is supported by an ancestral relationship between Chlamydiaceae, cyanobacteria and the chloroplast [82].

*On the origin of the nuclear envelope*. This scenario also suggests that the nuclear envelope developed concomitantly with the establishment of the mitochondria. This is in agreement with the proposal that the former developed to protect the genetic material from intracellular reactive oxygen species [83,84]. The concomitant gain of mitochondria and nuclear envelope was the final step in the evolution of the LECA and marked the birth of the eukaryotic domain by the gain of both of its trademark organelles.

*Gradual evolution of the archaea and eukaryotes*. There is substantial support for the hypothesis that the proto-eukaryote lineage accumulated numerous derived characters before the occurrence of the endosymbiotic event that led to the development of mitochondria [9]. Recent structural analysis of the protein repertoire also suggests that archaea began to evolve early but were established late [8]. Indeed, such an early start and late establishment of the three domains, as predicted by this scenario, would explain most of the controversy observed in the field.

*Other eukaryotic and archaeal features*. Of course, eukaryotes and archaea are not differentiated from bacteria by only the few characteristics listed here. In the LPCA scenario, this list is only the ‘tip of an evolutionary iceberg’ and more eukaryotic and archaeal characteristics are likely to be found upon further detailed characterization of the PVC members and once more powerful detection tools are developed.

*Falsifying the hypothesis*. The presented assumptions and derived scenarios are now open for falsification. The origin of the PVC features must be determined and the presence of additional eukaryotic- or archaeal-related ones should be investigated. Clear demonstration of HGT for the majority of the archaeal or eukaryotic features would falsify the proposed scenarios. A well-resolved phylogenetic tree of the PVC and of this superphylum into the bacterial one is one of the next important issues to be solved. A more complete characterization of the known and unknown PVC members will undoubtedly yield further insight. In addition, the development of PVC-specific biomarkers would allow the screening of the geological record, and endocytosis should be explored in archaea.

## 4. Conclusions

PVC members present particular features that are likely to have been present in their common ancestor and are usually associated with eukaryotes, archaea or both. Those features demonstrate the existence of intermediate steps between the three domains of life, discarding the need for fusion to explain eukaryogenesis and suggest that the LUCA was complex. We favour a scenario where the features found in PVC members are related to a bacterial ancestor, the LPCA, a sister group of the LAECA. The ‘cauldron hypothesis’ for the origin of eukaryotic and archaeal features supports a fuzzy framework of emergence. A bacterial ancestry of the LAECA provides for gradual evolution, internalization of the bacterial periplasm as the origin of the eukaryotic endomembrane system, including the nuclear envelope and the acquisition of mitochondria.

Although division of the ToL into three domains remains the norm, the PVC superphylum blurs the distinction between them, suggesting continuity between the three domains of life. Like the platypus is informative about the development of birds, reptiles and mammals, the current PVC members might provide a look at the very earliest steps in the archaeal and eukaryotic realms.
